# Roles of HMGB1 in regulating myeloid-derived suppressor cells in the tumor microenvironment

**DOI:** 10.1186/s40364-020-00201-8

**Published:** 2020-06-16

**Authors:** Shuiling Jin, Zhenzhen Yang, Xin Hao, Wenxue Tang, Wang Ma, Hong Zong

**Affiliations:** 1 Department of Oncology, the First Affiliated Hospital of Zhengzhou University, NO.1 Eastern Jianshe Road, Zhengzhou, 450052 Henan China; 2 Academy of medical science, Zhengzhou University, Zhengzhou, 450052 Henan China; 3 Henan college of Health Cadres, Zhengzhou, 450008 Henan China; 4 Departments of Otolaryngology, The Second Affiliated Hospital of Zhengzhou University, Zhengzhou, 450000 Henan China; 5 Center for Precision Medicine of Zhengzhou University, Zhengzhou, 450052 Henan China; 6 Henan Institute of Medical and Pharmaceutical Sciences, Zhengzhou University, NO.40 North Daxue Road, Zhengzhou, 450052 Henan China

**Keywords:** Myeloid-derived suppressor cells, Tumor microenvironment, High mobility group box 1

## Abstract

Myeloid-derived suppressor cells (MDSCs) are notable contributors to the immunosuppressive tumor microenvironment (TME) and are closely associated with tumor progression; in addition, MDSCs are present in most patients with cancer. However, the molecular mechanisms that regulate MDSCs in the etiopathogenesis of human tumor immunity remain unclear. The secreted alarmin high mobility group box 1 (HMGB1) is a proinflammatory factor and inducer of many inflammatory molecules during MDSC development. In this review, we detail the currently reported characteristics of MDSCs in tumor immune escape and the regulatory role of secreted HMGB1 in MDSC differentiation, proliferation, activity and survival. Notably, different posttranslational modifications of HMGB1 may have various effects on MDSCs, and these effects need further identification. Moreover, exosome-derived HMGB1 is speculated to exert a regulatory effect on MDSCs, but no report has confirmed this hypothesis. Therefore, the effects of HMGB1 on MDSCs need more research attention, and additional investigations should be conducted.

## Introduction

Carcinogenesis depends on inherent changes in the tumor microenvironment (TME) and inflammatory factors [1]. The inflammatory TME facilitates cancer progression, and an increasing number of reports have indicated that the TME exerts immunosuppressive effects, eliminating advantageous immune responses and harboring tumor cells. Accumulating evidence suggests that the most potent participant in immunosuppression is the population of immature myeloid cells (IMCs), also identified as myeloid-derived suppressor cells (MDSCs) [2, 3]. Studies have shown that MDSCs play an important role in tumor development, metastasis, and therapeutic resistance (including chemoresistance, radioresistance, and immunoresistance) [2, 4, 5]. However, the molecular mechanisms that regulate MDSCs in human cancer immunity remain unclear.

Existing research indicates that a variety of proinflammatory molecules drive MDSCs. The secreted alarmin high mobility group box 1 (HMGB1) is a proinflammatory partner, inducer and chaperone of many proinflammatory molecules involved in MDSC development [6]. HMGB1 was originally identified as a nuclear DNA-binding protein and performs multiple functions in the nucleus, including altering the DNA conformation to promote the binding of regulatory proteins, promote the integration of transposons into DNA, and stabilize the formation of nucleosomes [7]. However, the characteristics of HMGB1 as a secreted protein and an immunomodulator have been recognized only in the past 15 years [8]. In the following review, we focus on the introducing HMGB1 as an immunoregulator in the context of MDSC-mediated immunoregulation in the TME, and then provide additional possibilities for targeting MDSCs.

## MDSCs

MDSCs are a population of heterogeneous cells derived from bone marrow (BM) and have a significant inhibitory effect on immune cell responses [5]. In mice, MDSCs are marked by CD11b^+^Gr-1^+^ and can be subdivided into two different subsets: CD11b^+^Ly6G^+^Ly6C^low^ (polymorphonuclear MDSCs (PMN-MDSCs)) and CD11b^+^Ly6G^−^Ly6C^high^ (monocytic MDSCs (M-MDSCs)). In cancer patients, PMN-MDSCs are primarily defined by their CD11b^+^CD14^−^CD15^+^/CD66b^+^ phenotype, while M-MDSCs are characterized as CD11b^+^CD15^−^CD14^+^HLA-DR^−/low^. Notably, in humans, M-MDSCs can be isolated from monocytes based on the expression of the MHC class II molecule HLA-DR. However, to date, the only method that allows the separation of human PMN-MDSCs from neutrophils is gradient centrifugation using a standard Ficoll gradient. PMN-MDSCs are rich in low-density components, while neutrophils are rich in high-density components [5, 9]. Studies exploring the distinction between human PMN-MDSCs and neutrophils are ongoing, and it has been identified that lectin-type oxidized LDL receptor 1 (LOX-1) can differentiate human PMN-MDSCs from neutrophils more accurately, although not completely [10, 11].

The most important feature of MDSCs is their involvement in immune escape, which in turn promotes tumor progression [12]. On the one hand, MDSCs can produce high levels of immunosuppressive molecules, such as arginase 1 (ARG1), iNOS, TGFβ, IL-10, COX2, and indoleamine 2,3-dioxygenase (IDO), to immediately inhibit effector T cell-mediated cytotoxicity to tumor cells. New evidence shows that MDSCs can also suppress immune response mechanisms by inducing regulatory T cells (Tregs) [13–15], promoting macrophage polarization toward the M2 phenotype and differentiation into tumor-associated macrophages (TAMs) [16, 17], enhancing T helper 17 cell (Th17) differentiation [14], and inhibiting NK [18, 19] and B cell [20] immune activity. On the other hand, MDSCs can also promote tumor angiogenesis and epithelial-mesenchymal transition (EMT) by secreting molecules such as vascular endothelial growth factor (VEGF), TGFβ, and IL10 [21–23]. Furthermore, MDSCs can enhance stem-like propertie of breast cancer cells by affecting the IL-6/STAT3 and NO/NOTCH signaling pathways [24].

In the tumor immune microenvironment, the expansion and activation of MDSCs are induced by diverse cytokines produced by various cell types, including tumor cells, immune cells, and stromal cells, through various pathways. These cytokines can be subdivided into two categories: (A) Cytokines associated with the expansion of MDSCs. Published studies have identified many molecules, including granulocyte macrophage colony stimulating factor (GM-CSF), granulocyte colony stimulating factor (G-CSF), macrophage colony stimulating factor (M-CSF) and VEGF, that greatly influence MDSC expansion [12, 25]. Transcription factors such as STAT3 [26, 27], IRF8 [28, 29], and NOTCH [30, 31] play vital roles in the stimulation of these molecules. (B) Cytokines essential for MDSC activation, including IFN-γ, IL-1β, TNF, IL-4, IL-6, IL-13, and HMGB1. These cytokines signal mainly through the NF-κB [32], STAT1 [33], and STAT6 [34] pathways. Moreover, the oxidative phosphorylation [35] and glycolysis [36] pathways are related to the immunosuppressive function of MDSCs. Recently, endoplasmic reticulum (ER) stress has been considered to be related to the fate of MDSCs [37]. ER stress can enhance splenic PMN-MDSC apoptosis by activating the TNF-related apoptosis-induced ligand receptor (TRAIL-R)/caspase-8 pathway. This proapoptotic mechanism can further promote the expansion of MDSCs in the BM [38].

## HMGB1

**Fig. 1:**
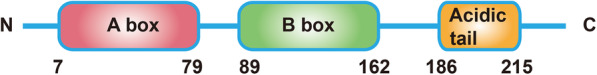
The structure of HMGB1 protein. The HMGB1 protein has two DNA-binding HMG-box domains; the N-terminal A box and central B box, and an acidic C-terminal tail

HMGB1 is a nonhistone chromosome-binding protein containing a single-chain polypeptide of 215 amino acids. HMGB1 has two DNA-binding HMG-box domains (the N-terminal A domain and central B domain) and an acidic C-terminal tail (Fig. 1). The B box is a functional domain for inflammatory activation; and the A box is the antagonist site of the B box and can thus block the inflammatory effect of the B box [7]. In most cells, HMGB1 is localized in the nucleus, where it acts as a DNA chaperone to help maintain nuclear homeostasis. However, under exposure to endogenous or exogenous stimuli, such as inflammatory cytokines, hypoxia, and stress, the HMGB1 protein can be actively secreted by cells. Alternatively, after the cell membrane integrity of necrotic or damaged cells is destroyed, HMGB1 is passively released from the cell. However, due to its lack of signal peptide sequences, HMGB1 is secreted not via classical protein secretion but via nonclassical secretion mediated by lysosomes. HMGB1 released from the cell as a damage-associated molecular pattern (DAMP) can act as a cytokine or chemokine; it can bind to cell surface receptors such as RAGE, TLR2, and TLR4, thereby activating inflammatory cells and promoting their proliferation and functional maturity, in turn increasing their ability to respond to chemokines [7, 39, 40].

The alarmin HMGB1 is released by most types of tumor cells and is detected in the serum of many cancer patients [40–44]. HMGB1 is ubiquitous in the TME and can interact with a variety of inflammatory cells to produce different results. HMGB1 derived from dead cells after chemotherapy or radiotherapy can induce the release of cytokines and the maturation of antigen-presenting cells. Moreover, HMGB1 can increase the release of CXCL12 from stromal cells and induce the aggregation of a large number of neutrophils and dendritic cells (DCs) at the tumor site, thereby eliminating infiltrating tumor cells [45]. On the other hand, as a tumor-promoting factor, HMGB1 is released by tumor cells and can increase the recruitment of immunosuppressive cells, thereby promoting tumorigenesis, invasion and metastasis. For example, CXCL12 induces recruitment of TAMs after forming a complex with HMGB1 under the influence of CXCR4 [7], and esophageal squamous cell carcinoma-derived exosomes (EXOs) promote PD1^+^ TAM expansion via HMGB1 [46]. HMGB1 promotes the expression of lymphotoxin α1β2 in tumor-infiltrating T cells, which can further lead to the recruitment of CD11b^+^F4/80^+^ macrophages to the tumor site. Macrophages secrete additional growth and angiogenic factors to further promote tumor growth [47]. Tumor cell-derived HMGB1 suppresses naturally acquired CD8^+^ T cell-dependent antitumor immunity by stimulating Tregs to produce IL-10, which is necessary for Treg-mediated immunosuppression [48].

**Fig. 2:**
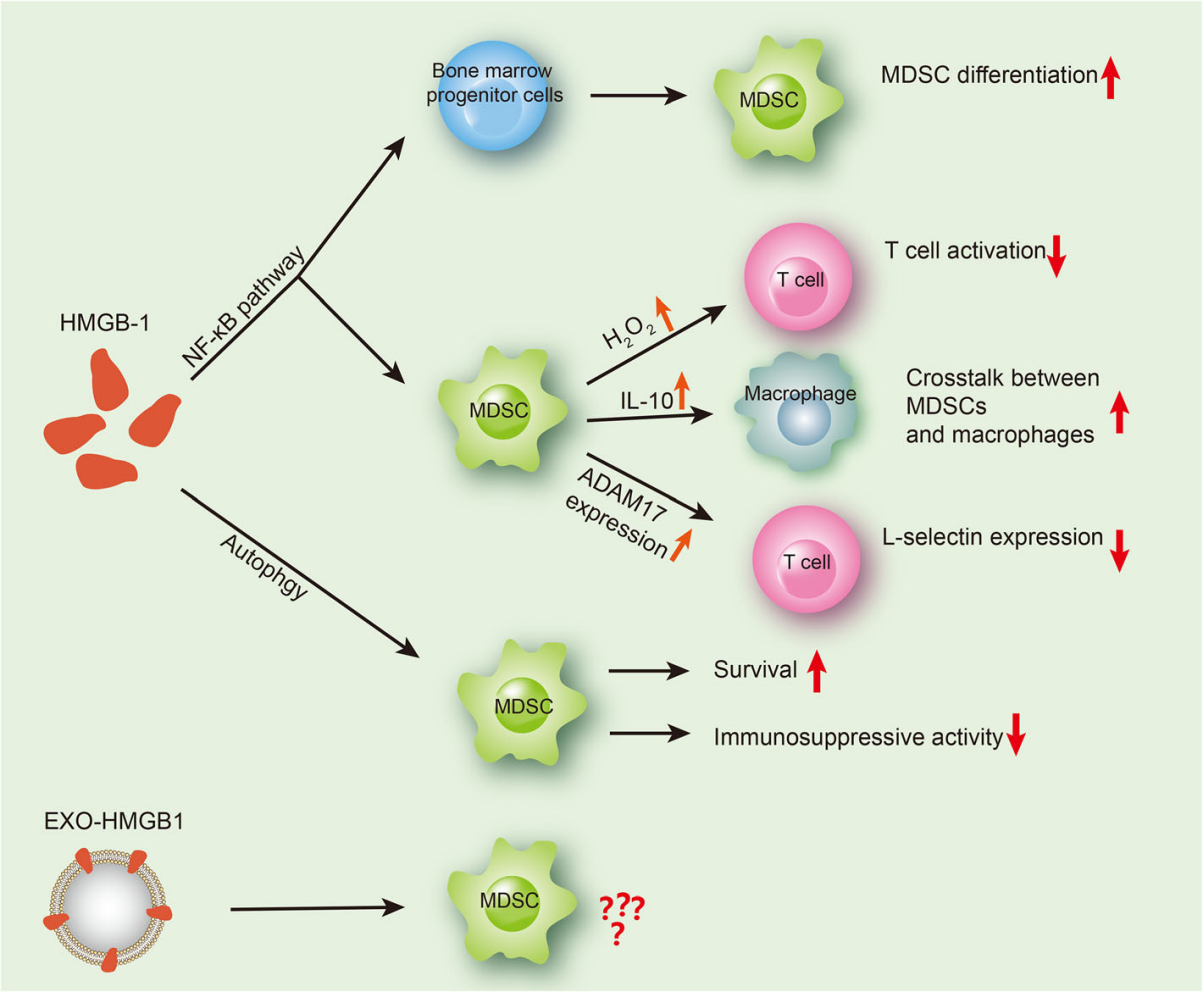
HMGB1 regulates MDSCs in the TME. EXO-HMGB1: exosomal HMGB1. In the TME, secreted HMGB1 mediates MDSC activation through NF-κB, thereby promoting the MDSC development from BM progenitor cells, contributing to MDSC-mediated T cell suppression, enhancing the crosstalk between MDSCs and macrophages, and downregulating the expression of the T cell homing receptor L-selectin. In addition, HMGB1 can mediate the survival of MDSC through autophagy while inhibiting the immunosuppressive activity of MDSC. Currently, the regulatory effect of EXO-HMGB1 on unclear is unclear and needs further exploration

As an important immunosuppressive cell type in the TME, MDSCs have been widely studied for their regulatory mechanism in various tumor types [2, 4]. Some reports have identified HMGB1 as a regulator of MDSCs, but the understanding is far from sufficient [6]. This review focuses on the main functions and effects of HMGB1 on the differentiation, survival, and activation of MDSCs, with an aim to increase the understanding of the mechanisms by which HMGB1 regulates immunosuppressive functions of MDSCs (Fig. 2).

## Regulation of MDSCs by HMGB1 in the TME

### HMGB1 contributes to the MDSC differentiation, activation and recruitment

Because HMGB1 is an important regulator of the TME, its effect on MDSCs has been studied, and it has been found to promote the differentiation, activation and chemotaxis of MDSCs. These functions are discussed in this section.

Li et al. provided the first report that secreted HMGB1 promotes the recruitment effect of MDSCs [49]. This group found that in a colon cancer mouse model, abdominal surgical trauma induced the release of large amounts of HMGB1 into the abdominal cavity, contributing to the recruitment of MDSCs into the abdominal cavity and promoting the formation of peritoneal metastases. This result suggests that minimizing surgical trauma during surgical colon tumor resection may be beneficial for preventing postoperative peritoneal metastasis and that intervention with the immune microenvironment after surgical trauma may help suppress peritoneal relapse of colon cancer [49]. HMGB1Ab was found to have a tumor-suppressive effect in a Renca tumor-bearing mouse model [50]. Both the differentiation and proliferation of MDSCs are controlled, and the inhibition ratio is positively correlated with the degree of HMGB1 downregulation. In vivo, the ability of HMGB1 to promote tumor development is seriously diminished as MDSCs are exhausted viaanti-Gr-1 antibodies. Therefore, this finding suggests that HMGB1 may mediate tumor immune escape by promoting the proliferation of MDSCs, a possibility that provides providing a new theoretical basis for preventing HMGB1-induced progression of renal cell carcinoma [50]. In addition, resveratrol-treated Lewis lung cancer cells express less HMGB1 and induce lower levels of less MDSC mobility than their untreated counterparts. This effect was partially reversed by treatment with exogenous recombinant HMGB1 [51]. In melanoma patients, the decreased number of eosinophils and the increased number of M-MDSCs as well as the increased baseline serum levels of the related inflammatory factors S100A8/A9 and HMGB1 indicate a lack of response to ipilimumab treatment [52].

Previous research suggested that tumor cells are the major contributors to HMGB1 secretion in the TME [53]. Parker et al. further identified the role and mechanism of HMGB1 in the functional regulation of MDSCs [32], showing that HMGB1 activates MDSCs through the NF-κB signaling pathway, increases the differentiation of MDSCs from BM progenitor cells, contributes to MDSC-mediated T cell suppression by increasing H_2_O_2_ production by MDSCs, enhances the crosstalk between MDSCs and macrophages by increasing IL-10 of MDSCs, and promotes the ability of MDSCs to downregulate the expression of the homing receptor L-selectin in naive T cells by sustaining ADAM17 expression in MDSCs [32]. HMGB1 binds to both TLR4 and RAGE, and the TLR4 and RAGE signals converge at NF-κB [7, 54]; in addition, MDSCs express both of these receptors [55, 56]. In the study by Parker et al. [32], the RAGE antagonist A Box partially restored T cell expression of L-selectin, suggesting that this effect of MDSCs may be regulated by RAGE. Both an HMGB1 inhibitor and an NF-κB inhibitor reduced IL-10 production by MDSCs during MDSC-macrophage crosstalk and inhibited the differentiation of MDSCs from BM progenitor cells; moreover, the NF-κB inhibitor restored T cell activation in the presence of MDSCs. Therefore, whether HMGB1 acts through TLR4 or through RAGE cannot be distinguished. However, regardless of which receptor is utilized, we can conclude that HMGB1 is a potent inducer of MDSCs and immunosuppression. The research by Parker et al. has identified that HMGB1 can enhance MDSC differentiation from BM cells and enhance the immunosuppressive activity of MDSCs [32]. However, questions remain: Does HMGB1 preferentially drive G-MDSC or M-MDSC differentiation? How does HMGB1 promote MDSC and BM cell differentiation? How do the different posttranslational modification states of HMGB1 affect the function of MDSC? These questions require further exploration by more researchers. In addition, the above observations focus on the direct regulatory effects of HMGB1 on MDSC; however, we believe that indirect effects also occur. For example, Tang et al. discovered an indirect effect, suggesting that HMGB1 stimulates the production of IL-23 in mouse melanoma in a RAGE-dependent manner, IL-23 promotes the expression of IL-17 produced mainly by γδT cells, and increased IL-17 levels can promote MDSC aggregation and tumor angiogenesis in mouse tumor tissue [57]. However, we believe that additional indirect mechanisms exist and require exploration in order to improve our fundamental understanding of HMGB1 in immunoregulation.

### HMGB1 affects MDSC survival through autophagy

Most studies have revealed that HMGB1 is an inducer for tumor cell autophagy and promotes tumor cell survival through autophagy [58–61]. Although the influence of autophagy on tumor cell survival has been well characterized, the link between MDSCs and autophagy remains poorly understood. To determine whether autophagy regulates the survival of MDSCs, Parker et al. cultured MDSCs under starvation conditions and found that the application of an autophagy inhibitor (chloroquine or bafilomycin) significantly decreased the proportion of surviving MDSCs, suggesting that autophagic MDSCs can increase the MDSC survival rate [62]. Other in vitro studies with autophagy inhibitors and HMGB1 inhibitors indicated that HMGB1 maintains MDSC viability by accelerating autophagy. Subsequent in vivo experiments utilizing 4 T1 breast tumor models showed that the autophagic ability of tumor-infiltrated MDSCs in tumor-bearing mice is more pronounced than that of MDSCs circulating in the peripheral blood. Collectively, these findings provide evidence that HMGB1 accelerates MDSC survival in the TME by inducing autophagy [62]. However, although it maintains the survival of MDSCs, autophagy also specifically reduces the immunosuppressive ability of MDSCs against CD4^+^ T and CD8^+^ T cells [62]. This effect may occur because autophagy enables cells to maintain their metabolic activity via the reuse of degraded nonessential cytoplasmic components, while autophagic survival eliminates some unnecessary cellular functions. Since tumor-infiltrated MDSCs are generally accepted to have a stronger inhibitory function than circulating MDSCs [4], this effect seems to contradict the autophagy-induced reduction in MDSC function. This apparent inconsistency may be attributed to other factors in the TME that drive MDSC efficacy and extend beyond the effects of autophagy on MDSC function.

### HMGB1 may exert its effects on MDSCs through EXOs

EXOs are small membrane-bound vesicles that are actively secreted by cells and contain a variety of biological substances, including miRNAs, circRNAs, proteins, lipids and soluble factors; these contents can be transferred between target cells, thereby playing important roles as mediators of intercellular communication [63]. By secreting EXOs, tumor cells can transfer immunostimulatory or immunosuppressive signaling molecules, thereby regulating the development, maturation and antitumor ability of the target immune cells [64]. Recently, HMGB1 has been shown to be expressed on tumor-derived exosomal membranes [46]. EXOs derived from liver cancer cells induce B cells to differentiate into TIM-1^+^ Bregs via the HMGB1-TLR2/4-MAPK pathway, and this increased infiltration of TIM-1^+^ Bregs is related to advanced disease and poor survival in patients with liver cancer [65]. Gastric cancer cell-derived EXOs carry HMGB1 and promote the migration of gastric cancer cells by inducing neutrophil autophagy through TLR4/NF-κB [66]. Tumor-derived EXOs play a key role in the expansion, survival, and immunosuppressive effects of MDSCs [67]. Melanoma-derived EXOs can promote the differentiation of BM cells into CD14^+^HLA-DR^−^ cells that secrete TGF-β while inhibiting the differentiation of BM cells into DCs [68]. Functional analysis has shown that tumor-derived EXOs can induce MDSC polarization toward the M2 phenotype and accelerate the Th2 immune response. In addition, tumor-derived EXOs can promote MDSC survival by enhancing the expression of the antiapoptotic protein Bcl-xL and activating the STAT1/3 pathway [69]. Thus, we speculate that HMGB1 in tumor-derived EXOs may also affect on the proliferation, differentiation, or migration of MDSCs, although no related reports have been published to date. Therefore, additional research on EXOs is needed to be explored to clarify the role of exosomal HMGB1 in MDSC regulation.

## HMGB1 regulates MDSCs in nonneoplastic diseases

MDSCs are important participants in inflammatory diseases, and HMGB1 can also regulate MDSCs in nontumor diseases. However, few related results have been published to date.

Functional changes after severe trauma have a focus of immunological research in patients and animal models [70]. Ruan et al. showed for the first time in mice, that an HMGB1-neutralizing antibody ameliorates the weakened T cell response and reduces the population of CD11b^+^Gr-1^+^ MDSCs in the spleen two days after peripheral tissue trauma [71]. Therefore, the anti-HMGB1 antibody strategy deserves further evaluation as a method for reducing infection and multiple organ dysfunction after trauma.

HMGB1 is released from the ischemic brain during the hyperacute phase of stroke in patients and mice. Cytokines peripherally secreted in response to brain injury induce disease behavior. However, the subsequent release of HMGB1 induces the efflux of MDSCs from the BM and the proliferation of MDSCs in the spleen, thereby suppressing the adaptive immune response. In addition, HMGB1-RAGE signaling leads to failure of mature monocytes and to lymphopenia. This study describes the HMGB1-RAGE-mediated pathway as a key mechanism that explains complex brain-immune interactions after ischemia [72].

## Conclusions

Here, we noted that HMGB1 can regulate the differentiation, activation and survival of MDSCs through various pathways. However, many issues remain to be explored: 1. How does HMGB1 affect MDSCs in different tumor types or in different growth stages of the same tumor type? 2. HMGB1 was reported to negatively affect the immunoregulatory activity of MDSCs through autophagy, but the same group previously reported that HMGB1 contributes to the tolerance of MDSCs. Since HMGB1 is not the only factor that affects the survival and function of MDSCs, additional research is needed to accurately understand the interaction between the transition of tumor MDSCs and their inhibitory activity. 3. Since HMGB1 can be modified by posttranslational modifications, it is important to assess whether HMGB1 modifications (phosphorylation, nitrosation, glycosylation, etc.) affect the function and survival of tumor MDSCs. 4. The regulatory effect of exosomal HMGB1 on MDSCs has not been confirmed; thus, additional exploration is needed to clarify the effect of HMGB1, as exosomal cargo, on MDSCs and determine the difference between secreted and exosomal HMGB1 in MDSC regulation.

## Data Availability

The dataset supporting the conclusions of this article is included within the article.

## Abbreviations

TME: Tumor microenvironment
IMCs: Immature myeloid cells
MDSCs: Myeloid-derived suppressor cells
PMN-MDSCs: Polymorphonuclear MDSCs
M-MDSCs: Monocytic MDSCs
DCs: Dendritic cells
Tregs: Regulatory T cells
TAMs: Tumor-associated macrophages
HMGB1: High mobility group box 1
LOX-1: Lectin-type oxidized LDL receptor 1
ARG1: Arginase 1
IDO: Indoleamine 2, 3-dioxygenase
EMT: Epithelial-mesenchymal transition
GM-CSF: Granulocyte macrophage colony stimulating factor
G-CSF: Granulocyte colony stimulating factor
M-CSF: Macrophage colony stimulating factor
VEGF: Vascular endothelial growth factor
TRAIL-R: TNF-related apoptosis-induced ligand receptor
DAMP: Damage-associated molecular pattern
RAGE: Receptor for advanced glycation end products
EXOs: Exosomes
